# Development of a Novel Large Animal Model to Evaluate Human Dental Pulp Stem Cells for Articular Cartilage Treatment

**DOI:** 10.1007/s12015-018-9820-2

**Published:** 2018-05-04

**Authors:** Tiago Lazzaretti Fernandes, Kazunori Shimomura, Andre Asperti, Carla Cristina Gomes Pinheiro, Heloísa Vasconcellos Amaral Caetano, Claudia Regina G. C. M. Oliveira, Norimasa Nakamura, Arnaldo José Hernandez, Daniela Franco Bueno

**Affiliations:** 1 Institute of Orthopedics and Traumatology, Hospital das Clínicas, School of Medicine, University of São Paulo, 333 Dr. Ovídio Pires de Campos, São Paulo, 05403-010 Brazil; 2 Hospital Sírio-Libanês, 115 Rua Dona Adma Jafet, Bela Vista, São Paulo / SP, 01308-050 Brazil; 3 Department of Orthopedics, Osaka University Graduate School of Medicine, 2-2 Yamadaoka, Suita, Osaka, 565-0871 Japan; 4 Center for Advanced Medical Engineering and Informatics, Osaka University, 2-2 Yamadaoka, Suita, Osaka, 565-0871 Japan

**Keywords:** Mesenchymal stem cells, Tissue engineering, Human dental pulp stem cells, Articular cartilage, Hyaline cartilage, pre-clinical study, large animal model, miniature pig

## Abstract

**Purpose:**

Chondral lesion is a pathology with high prevalence, reaching as much as 63% of general population and 36% among athletes. The ability of human Dental Pulp Stem Cells (DPSCs) to differentiate into chondroblasts *in vitro* suggests that this stem cell type may be useful for tissue bioengineering. However, we have yet to identify a study of large animal models in which DPSCs were used to repair articular cartilage. Therefore, this study aimed to describe a novel treatment for cartilage lesion with DPSCs on a large animal model.

**Methods:**

Mesenchymal stem cells (MSC) were obtained from deciduous teeth and characterized by flow cytometry. DPSCs were cultured and added to a collagen type I/III biomaterial composite scaffold. Brazilian miniature pig (BR-1) was used. A 6-mm diameter, full-thickness chondral defect was created in each posterior medial condyle. The defects were covered with scaffold alone or scaffold + DPSCs on the contralateral side. Animals were euthanized 6 weeks post-surgery. Cartilage defects were analyzed macroscopically and histology according to modified O’Driscoll scoring system.

**Results:**

Flow cytometry confirmed characterization of DPSCs as MSCs. Macroscopic and histological findings suggested that this time period was reasonable for evaluating cartilage repair. To our knowledge, this study provides the first description of an animal model using DPSCs to study the differentiation of hyaline articular cartilage *in vivo*.

**Conclusion:**

The animals tolerated the procedure well and did not show clinical or histological rejection of the DPSCs, reinforcing the feasibility of this descriptive miniature pig model for pre-clinical studies.

## Introduction

Chondral lesion is a pathology with high prevalence, reaching as much as 63% of general population and 36% among athletes, causing important clinical repercussions and decrease in quality of life [1–3].

Defects of the cartilage, when left untreated, may enlarge and result in lesions in the underlying subchondral bone, leading to biomechanics and homeostasis disturbances in the knee as a whole. This process may result in loss of mobility, wear and arthritis [4].

Osteoarthritis (OA) is estimated to occur in up to 22.7% of the United States population and it has been forecast that more than 50 million people will be affected by OA by the year of 2020. It will be a major cause of morbidity and physical limitation among individuals older than 40 years [5].

Cartilage is a unique avascular and aneural tissue that does not readily regenerate once damaged because blood contains a limited number of the growth factors required for cellular healing [6]. Despite the numerous techniques available today, complete healing of damaged or defective cartilage and the consistent reproduction of normal hyaline cartilage is not possible. For these reasons, continuous drug therapies and secondary surgeries are common, and new therapeutics for articular cartilage lesions is of elevated clinical relevance [7].

Mesenchymal stem cell-based therapy has received considerable research attention because of the relative ease of handling the tissue harvest and subsequent cell expansion and differentiation [8].

Therefore, stem cell therapy is a promising option to facilitate regenerative tissue repair [8, 9] . These cells may be isolated from various known tissues, such as bone marrow, adipose tissue, and the synovial membrane [8].

Recently, dental pulp has been shown to contain a stem cell niche and these dental pulp stem cells (DPSCs) maintain their self-renewal capacity due to the active environment in the dental pulp of deciduous [10, 11]. Furthermore, this stem cell niche protects DPSCs from the cumulative effects of genetic and environmental factors [11].

DPSCs are characterized as mesenchymal stem cells (MSCs) based on their plastic adherence and the expression of specific stem cell markers, such as CD29, CD90, CD44 and Stro-1 [10–12].

DPSCs show higher proliferative and immunomodulatory capacities than bone marrow MSCs (BM-MSCs) because they retain multilineage differentiation capacity and they are collected from extracted third molars, deciduous teeth and other healthy teeth [13, 14].

The ability of DPSCs to differentiate into chondroblasts and osteoblasts *in vitro* suggests that this stem cell type may be useful for tissue bioengineering to treat bone and cartilage injuries [12].

Although DPSCs have shown promising results and have been used to treat osseous defects, we have yet to identify any animal models in which DPSCs were used for articular cartilage repair that would provide a basis for further use in clinical practice [10].

It is widely accepted that MSCs exhibit immune-tolerance capacity and the availability of allogenic MSCs to repair cartilage lesions has been reported in animal model [15].

As shown in previous studies, the miniature pig is a suitable large animal model for the preclinical testing of different treatments [16, 17]. In Brazil, the miniature pig BR-1 is the only Brazilian miniature pig that had been exclusively developed for research [18].

In summary, articular cartilage is at high risk of damage during initial trauma and development of osteoarthritis is estimated to cause important physical limitations. Investigation of a novel cartilage treatment with MSCs derived from dental pulp deserves a large animal model for translational studies.

### Purpose

The purpose of this study was to describe and validate the feasibility of using a large animal model (Brazilian miniature pig) to treat articular cartilage with human DPSCs.

## Methods

Human origin strain was collected from a 9 years-old boy deciduous tooth in a dentistry office. Cells were isolated and cultivated in a Good Manufacturing Practice (GMP) Laboratory and, afterwards, used in the swine large animal model.

Two hundred thousand DSPCs (2 × 10^5^) were added to a biomaterial composite of bilayer collagen type I/III (Geistlich**®**, Wolhusen, Switzerland) and cultured at least for 24 h in 24-well plates with 4 ml of DMEM/F-12 supplemented with 15% FBS. The biomaterial containing DPSCs was maintained in a humidified atmosphere of 5% CO_2_ at 37 °C.

Composite adherence was similar to Costa et al. [10] and Bueno et al. [19] seed descriptions. From 2 × 10^5^ seeded cells in the biomaterial, we waited 2 h for the adhesion time of the cells to the biomaterial and then the biomaterial was transferred to another plate and cells were counted. Through this experiment, it was observed an efficiency rate of 96% cell adhesion to the biomaterial.

Afterward, the DSPC-laden biomaterial was sent to the operating room for use in the animal model.

Details of experimental animal model are written on methods, including the topics: animal protocol, surgical techniques, cartilage defects, and postoperative care and euthanasia.

### Characterization of MSCs from Dental Pulp Tissue

Human origin strain mesenchymal stem cells was obtained from deciduous tooth as mentioned before. The pulp was pulled from the teeth and washed twice with PBS (Gibco Invitrogen; pH 7.4, Grand Island, NY, USA). For digestion, 1 mg/ml trypsin (TrypLE, Gibco Invitrogen) was used for 30 min at 37 °C. Digestion was terminated by adding 4 ml Dulbecco’s Modified Eagle Medium/Nutrient Mixture F-12 (DMEM/F-12; Gibco Invitrogen) supplemented with 15% fetal bovine serum (FBS; Gibco Invitrogen) followed by centrifugation.

The samples were cut into appropriate fragment sizes. The fragments were plated into three or four wells of a 24-well plate. Fragments were maintained under a humidified atmosphere of 5% CO_2_ at 37 °C. After ten days, when the fragments had expelled DPSCs, the fragments were trypsinized using standard procedures at 80% confluence to expand the lineage.

### Characterization by Flow Cytometry

DPSC strains were characterized using flow cytometry. One hundred thousand cells (1 × 10^5^) from each population were used. The cells were stained with the following monoclonal antibodies: CD29-PE, CD31-FITC, CD34-FITC, CD44-PE, CD45-PE, CD73-FITC, CD90-FITC, CD105-PE and CD117-PE (Becton Dickinson, Franklin Lakes, NJ, USA). Appropriate isotype-matched control antibodies were used for all of the analyses. Cells were analyzed using a FACSCalibur flow cytometer with Cell Quest Software (Becton Dickinson).

### DPSC Scaffold Attachment – Electron Microscopy

For transmission electron microscopy, specimens were fixed with 2% glutaraldehyde for 2 h and post-fixed in 0.15 M phosphate buffered osmium tetroxide solution (pH 7.2) for 1 h at 4 °C. The samples were washed in a solution containing 1.2 g sodium chloride, 14.6 g saccharose and 200 ml distilled water and then stained with 1% uranyl acetate overnight at 4 °C. Dehydration was performed using acetone, and then the tissues were embedded in araldite. Ultrathin sections (50–70 nm) were stained with lead citrate and examined using a JEM 1010 electron microscope (JEOL USA, Peabody, MA, USA).

### Animal Protocol

Two male Brazilian miniature pigs (BR-1, Minipig Pesquisa e Desenvolvimento, Ltda., Campina do Monte Alegre, SP, Brazil) aged 8 and 12 months, and weighing 19 and 22 kg, respectively, were used for the experimental protocol.

Surgeries were performed on both side’s hind limbs of the BR-1 miniature pigs. Following cartilage defect generation, the first side received collagen type I/III scaffold only (Geistlich®, Wolhusen, Switzerland). After suturing the first side arthrotomy, the opposite side was operated. The second hind limb cartilage defect received scaffold collagen type I/III (Geistlich®, Wolhusen, Switzerland) seeded with DPSCs.

The animals were kept in bays with pine shavings (deep bedding method, Embrapa) and were fed twice a day with food and water ad libitum.

### Surgical Techniques

First, the animals received intramuscular ketamine and midazolam. The induction of general anesthesia was performed with propofol, and anesthesia was maintained throughout surgery with isoflurane. Morphine was used for analgesia. Mini-pig specimens were placed in the supine position on the operating table. Both lower extremities were scrubbed and sterilely draped.

Medial arthrotomies of both swine posterior knees (~5 cm) and partial resections of the fat pad were performed to expose the condyle articular cartilage and the anterior cruciate ligament [20].

#### Cartilage Defects

**Fig. 1:**
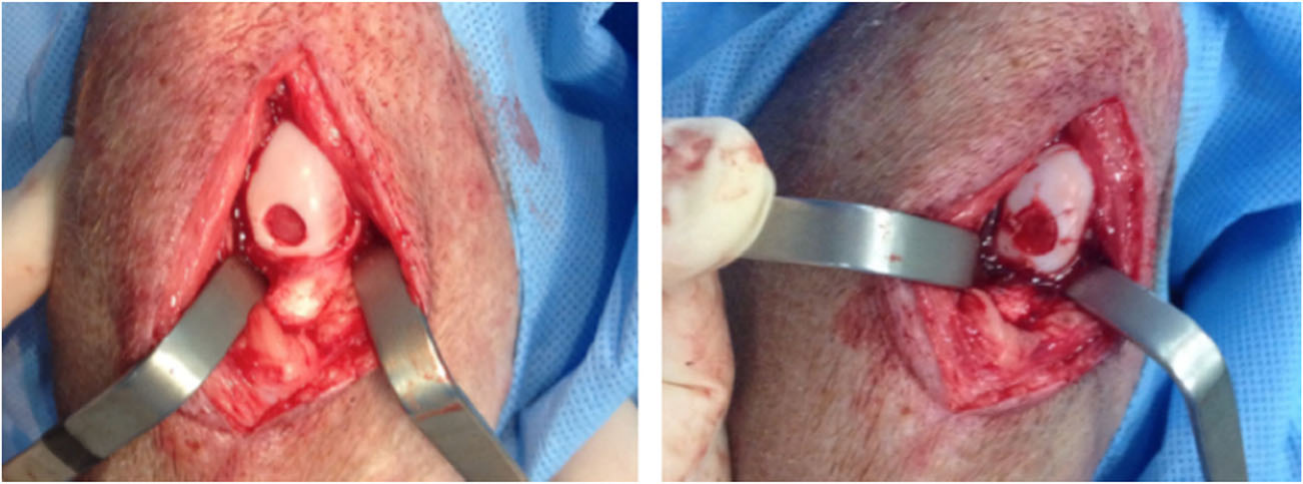
Left Side: Cartilage Defect (6-Mm Diameter) In The Inferior Femoral Condyle After Calcified Cartilage Layer Removal. Right Side: Scaffold With Mesenchymal Stem Cells Sutured Into Defect

A full thickness chondral defect measuring 6 mm in diameter was generated in a load-bearing area of the host animal’s medial femoral condyle in each posterior miniature pig limb using a biopsy punch, followed by removal of the calcified cartilage layer (Fig. 1). The full-thickness damaged cartilage was covered with the biomaterial scaffold alone in one limb and scaffold loaded with DPSCs in the other posterior limb (described above). The scaffold was stabilized with 6–0 vicryl sutures.

### Postoperative Care and Euthanasia

Animals were provided tramadol, meloxicam, dipirone and metamizole for postoperative pain control and cefazolin for infection prophylaxis. Weight bearing and nutrition were allowed ad libitum*.* The miniature pigs were able to weight-bear within 12 h and walked normally for up to 10 days.

At six weeks post-surgery, each animal was euthanized with an overdose of propofol and potassium chloride. The hind limbs were disarticulated at the hip.

### Histopathology

Osteochondral specimens were fixed in 10% formalin solution for 24 h. Decalcification was performed in 5% formic acid for 48 h, and specimens were embedded in paraffin wax. Sections (5 μm) were stained with hematoxylin and eosin and examined using an AxioVert II light microscope (Carl Zeiss, Oberkochen, Germany).

## Results

Present data shows protocol feasibility of Brazilian miniature pig BR-1 for cartilage evaluation after scaffold and DPSCs treatment.

### Characterization of MSCs from Dental Pulp Tissue

**Fig. 2:**
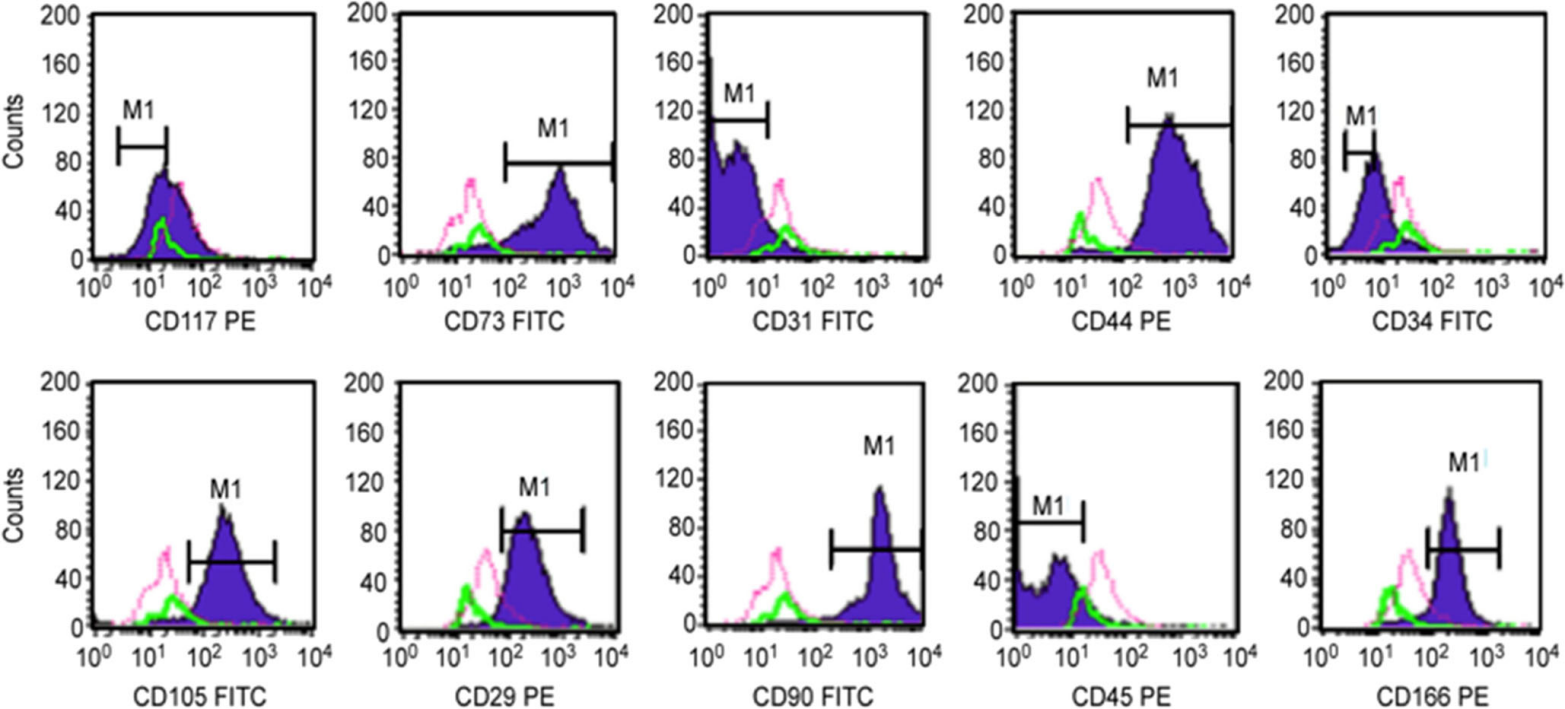
Flow Cytometry Analysis Showing Positive Reactions To Mesenchymal Markers (CD29, CD73, CD105, CD90, CD166 And CD44) And Negative Reactions To Hematopoietic (CD34 And CD45) And Endothelial Markers (CD31)

Flow cytometry analyses confirmed the characterization of DPSCs as MSCs (Fig. 2).

### DPSC Attachment to Scaffolds – Electron Microscopy

**Fig. 3:**
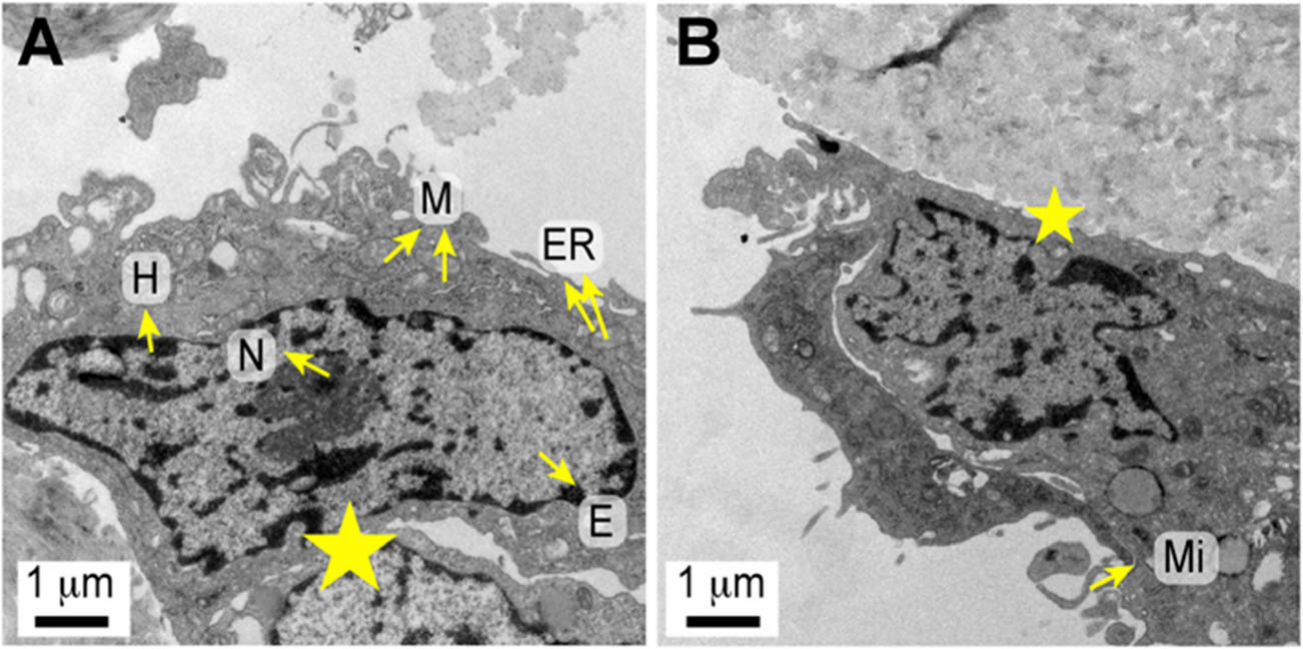
Transmission Electron Micrographs Of The Ultrastructure Of The Mesenchymal Stem Cells Derived From Dental Pulp Showing (**a**) Intracellular Organelles And Nuclear Morphology, Including Heterochromatin, Euchromatin, And Nucleolus (Original Magnification 15,000×). **b** Microvilli On The Cell Surface. Structural Surface Modifications Of Cell–Cell (a) And Cell–Extracellular Matrix Interactions At The Border Zone (b) (Star). (Original Magnification 15,000×) (Abbreviations: ER, Endoplasmic Reticulum; H, Heterochromatin; E, Euchromatin; Nu, Nucleolus; M, Mitochondria; Mi, Microvilli)

Transmission electron microscopy revealed that the MSCs derived from dental pulp had intact membranes and scattered microvilli-like structures on their surfaces, which might be related to their inherent adhesive capability. Cells extended many structurally flat surfaces for cell–cell and cell–extracellular matrix interactions at the border zone. The fibroblast-like MSCs showed a high nucleus-to-cytoplasm ratio. An indented ovoid-shaped nucleus was finely distributed with heterochromatin and euchromatin. The cytoplasm was rich in ribosomes and morphoplasms, which are membranous organelles containing a well-developed endoplasmic reticulum and Golgi apparatus as well as many mitochondria. Rough endoplasmic reticulum membranes with abundant ribosomes were scattered throughout the cytoplasm, suggesting the active synthesis of proteins required for cell proliferation and adhesion (Fig. 3).

### Macroscopic Evaluation

Tables 1 and 2 showed gold standard macroscopic and histological classification examples of hind limb comparison of the same miniature pig, respectively. Description purpose was to access animal model feasibility only. There was no intention to predict or to evaluate which treatment was better or equivalent, but novelty of DPSCs usage for articular cartilage treatment.

**Table 1:** Macroscopic scoring system example (ICRS macroscopic evaluation of cartilage repair [21]) – Miniature pig 1

Cartilage repair assessment ICRS		Score		
		Hyaline cartilage	Scaffold only	Scaffold + DPSCs
1. Degree of defect repair		4	1	3
In level with surrounding cartilage	4			
75% repair of defect depth	3			
50% repair of defect depth	2			
25% repair of defect depth	1			
0% repair of defect depth	0			
2. Integration to border zone		4	0	2
Complete integration with surrounding cartilage	4			
Demarcating border <1 mm	3			
3/4th of graft integrated, 1/4th with a notable border >1 mm width	2			
1/2 of graft integrated with surrounding cartilage, 1/2 with a notable border >1 mm	1			
From no contact to 1/4th of graft integrated with surrounding cartilage	0			
3. Macroscopic appearance		4	0	1
Intact smooth surface	4			
Fibrillated surface	3			
Small, scattered fissures or cracs	2			
Several, small or few but large fissures	1			
Total degeneration of grafted area	0			
Overall repair assessment		12	1	7
Grade I: normal	12			
Grade II: nearly normal	11–8			
Grade III: abnormal	7–4			
Grade IV: severely abnormal

**Table 2:** Histological scoring system example (Modified form O’Driscoll et al.) [22] – Miniature pig 1

Characteristics		Score		
		Hyaline cartilage	Scaffold only	Scaffold + DPSCs
1. Cell morphology		4	2	2
Hyaline like articular cartilage	4			
Partial differentiated hyaline cartilage	2			
Fibrous tissue	0			
2. Integrity of surface		3	2	2
Surface smooth and intact	4			
Surface horizontal fibrillation	2			
Surface shows fissures to 25–100% of the depth of the cartilage	1			
Serious deep interruption of the surface and many deep fibrillations	0			
3. Thickness		2	0	1
100% of normal host cartilage	2			
50–100% of normal cartilage	1			
0–50%	0			
4. Surface of area filled with cells		3	0	1
100–75%	3			
75–50%	2			
50–25%	1			
25–0%	0			
5. Chondrocyte clustering		2	2	2
None at all	2			
< 25% of the cells	1			
25–100% of the cells	0			
6. Degenerative changes		3	3	3
Normal cell quantity, no clustering, normal staining with proteoglycan specific stain	3			
Normal cell quantity, some cluster formation, moderate staining	2			
Clearly less cells, poor staining	1			
View cells, no or very little staining	0			
7. Restoration of the subchondral bone		4	2	2
Normal and straight	4			
Slight contour changes	2			
Larger interruptions in subchondral bone	1			
Defect	0			
8. Integration		2	2	2
Both sides of repair tissue integrated with host cartilage	2			
One side integrated	1			
No integration	0			
Total maximal score = 23		23	13	15

Scaffold only and scaffold seeded with DPSCs miniature pig 1 scores are shown on Tables 1 and 2.

Initial cartilage healing was observed in a macroscopic evaluation of both knees six weeks after treatment.

**Fig. 4:**
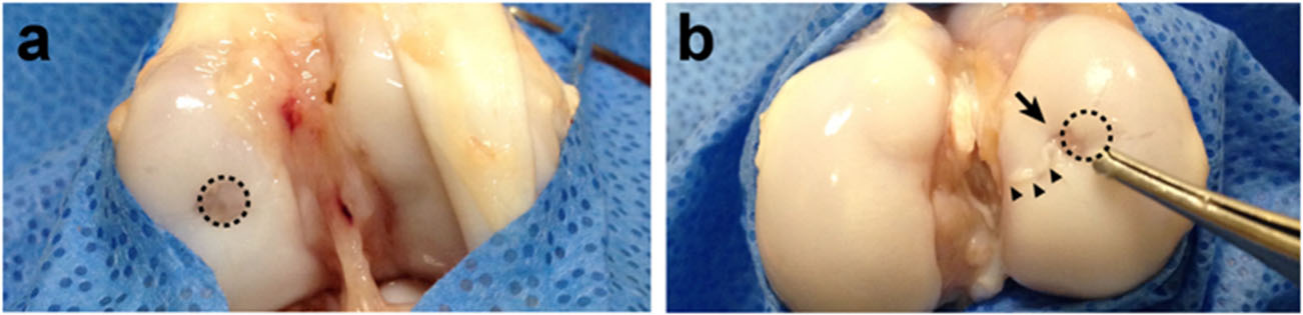
Macroscopic Examination Of Condyle Cartilage Defects After 6 Weeks In A Knee Treated With A Scaffold Containing No Dpscs (**a**) And A Scaffold Plus Dpscs (**b**). Dotted Circle: Area Of The Original Defect; Arrow: Irregular Coverage Of The Defect In The DPSC-Treated Sample; Arrowheads: Tissue Growth Over The Cartilage

The cartilage lesion seeded with scaffolds loaded with DPSCs exhibited irregular eccentric coverage of the defect and new tissue growth over the cartilage. Scaffold without stem cells on the opposite knee showed a regular defect border and shallow coverage tissue over the defect (Fig. 4).

### Histopathology

Similar to the macroscopic evaluation, remodeling was observed in both knees after six weeks.

The O’Driscoll modified score with 23 valid points presented on Table 2 showed similar values for the cartilage defect treated with scaffold alone: 13 points, and scaffold plus DPSCs: 15 points [22].

**Fig. 5:**
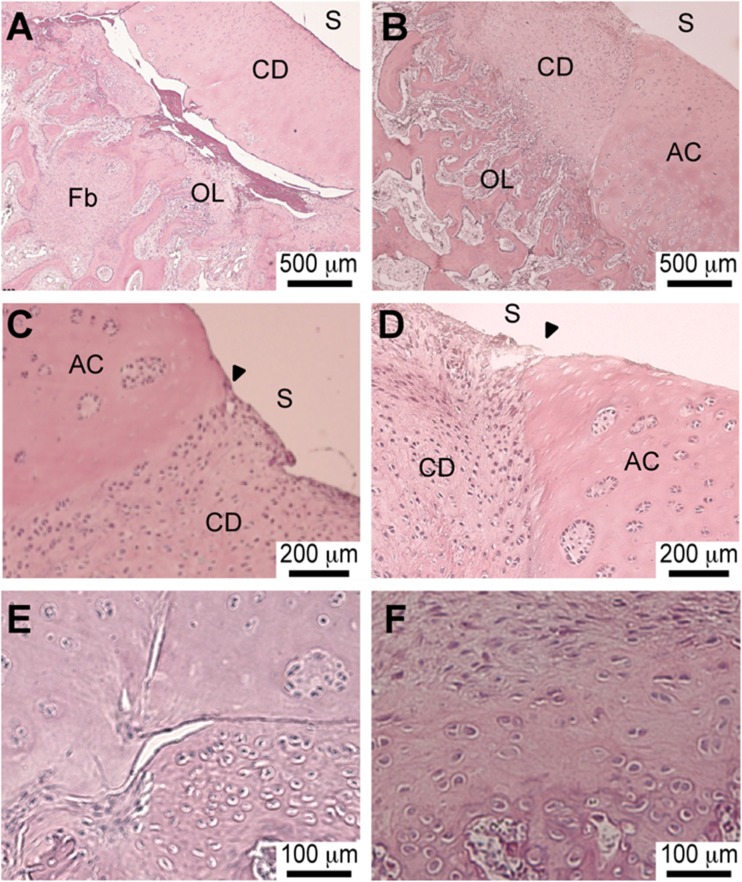
Histology Of The Scaffold Plus DPSC-Treated (**a**, **c** & **e**) And Scaffold-Treated Samples (**b**, **d** & **f**). A & B: Lower Magnification; C & D: Transition Zone; E & F: Cell Morphology. Legend: Narrow Head - Transition Zone; Surface (S); Adjacent Cartilage (AC); Critical Size Defect (CD), Endochondral Ossification Layer (OL); Fibroblastic Proliferation (Fb)

Microscopic findings of this miniature pig sample, that has to be confirmed in a larger animal study, showed an inflammatory response in the endochondral ossification layer of the scaffold plus DPSC-treated knee, with increased cellularity (Fig. 5).

Compared to scaffold alone, scaffold plus DPSCs showed a thicker deep layer. In addition, an increase in the fibroblastic tissue was observed on the superficial layer of the cartilage defect treated with scaffold alone (Fig. 5).

## Discussion

To our knowledge, this study provides the first description of an animal model using DPSCs to study the differentiation of hyaline articular cartilage *in vivo*.

Our first experiment and IRB approval of DPSCs and cartilage repair treatment dated from August 2015. We could observe in both DPSCs and collagen type I/III scaffold experimental group, and no-DPSCs scaffold only group, hyaline articular cartilage covering the defect with different layers of chondrocytes and extra-cellular matrix, from dense to shallow thickness compared to the adjacent hyaline articular cartilage from the edge of the defect (Fig. 5).

Hilkens et al. [13, 12] and Karbanová et al. [22, 23] each studied the general osteochondral differentiation of DPSCs *in vitro*; however, hyaline or articular cartilage was not studied [7].

The animals did not show clinical rejection of the DPSCs and tolerated the procedure well, reinforcing the feasibility of this mini-pig model.

Although the therapeutic potential of a treatment is typically first tested with *in vivo* experiments in small animals, large animal studies, particularly in swine or miniature pigs, are prerequisites for treatment validation before translation into clinical patients. This gradual transition to clinical patients is necessitated by the many key differences between small animal models and humans [24, 25].

Importantly, large animals, such as the mini-pig, have characteristics and dimensions similar to the human body, and they are also similar to humans in terms of platelet counts, clotting parameters, metabolic rates and bone structures [17, 18, 24].

The development of preclinical models in large animals requires the use of well-characterized animal cell lines similar to their human counterparts, and the miniature pig is a suitable model for it [16–18].

The isolation, cultivation, and differentiation of MSCs from Brazilian miniature pig BR1 makes this animal eligible for stem cell-based studies. Minipigs have close similarities to humans in terms of MSCs characteristics as morphology, proliferation, colony formation capacities and negative expression of MHC-II. Moreover, RT-PCR mRNA levels show high gene expression of COLII [18].

Cell therapy with mesenchymal stromal cells has been widely investigated in different animal models. Pelatti et al. [26] stated that xenogeneic cell transplantation, which was done without immunosuppression, was well tolerated in all animals with no apparent long-term adverse effect. They also presented that repeated heterologous stem-cell injection were a safe procedure [26].

In instance, Ankrum et al. [27] showed that culture-expanded MSCs express low levels of MHC class I and are negative for MHC class II. Unmatched allo- and xenogeneic-MSCs can preferentially persist within *in vivo* environments that are immune suppressed [27].

Related specifically to DPSCs in swine models, Tang et al. [28] showed that DPSCs failed to stimulate allogeneic T-cell proliferation. DPSCs show low immunogenicity and immunomodulatory activities on T lymphocytes, which may expand the source of and interest in DPSCs [28]. Costa et al. [10] also stated that they did not use any protocol for immunosuppression in experimental animals and no clinical symptoms of human DPSCs rejection were observed in the recipient animals.

The side-by-side procedures performed in this study have the advantage of allowing paired comparisons. Knees were exposed to the same intrinsic and extrinsic conditions, such as weight bearing, improving the study design.

This study simulated the physiological *in vivo* conditions of load and degenerative changes. Other authors have performed cartilage defect studies on non-weight-bearing zones, such as the trochlea [29, 30].

Lateral condyle cartilage lesions are a good reproduction of the biomechanical trauma observed during knee sprains and ACL lesions. However, the presence of the anterior tendon over the lateral condyle prevented optimal visualization of the working area in a minimally traumatic procedure.

DPSCs expressed MSC markers and electron microscopy revealed that exhibited good attachment to the biomaterial scaffold. Appropriate attachment is a key factor for tissue engineering, as the scaffolds establish a three-dimensional structure that retains the seeded cells and provides mechanical support to aid in cartilage development over time [7].

Because this single analysis may have internal bias and the reported findings do not represent group treatments, we would like to emphasize that the main contribution of this manuscript is the methodological description of a feasible large animal model to analyze the effects of DPSCs on cartilage lesions.

This study did not intend to compare treatments with and without DPSCs due to the risk of coincidental findings. Future studies assessing the effects of DPSCs on hyaline articular cartilage in large animal models may increase our knowledge and appear promising.

We agree that time period of 6 weeks is a short post surgery period. Nevertheless, our purpose was to evaluate feasibility of this miniature pig model and we recommend that long-term follow-up period should be expected for potential clinical usage and cartilage regeneration. Six weeks period was originally based on Costa et al. [10] protocol and results, which states for human DPSCs that regeneration process was more advanced one-month post surgery, and that the tissue appears to be denser and more mature [10].

Future studies are desired and have been conducted to compare different MSC lineages for articular cartilage treatment with more subjects. Finally, this translational animal model has promising applications on clinical practice. Once analyses confirm positive results, it should permit a subsequent clinical trial in humans.

### Conclusion

This preliminary report described a feasible Brazilian miniature pig model for the treatment of articular cartilage lesion using human DPSCs. The animals tolerated the procedure well and did not show clinical or histological rejection of the DPSCs.
